# Supplementary figures and images for: Evaluating how demography and temperature increase might alter the burden of congenital Toxoplasmosis in Africa
Source: PLoS Negl Trop Dis. 2026 Mar 6;20(3):e0014058. doi: 10.1371/journal.pntd.0014058 (PMC12974952; doi:10.1371/journal.pntd.0014058)

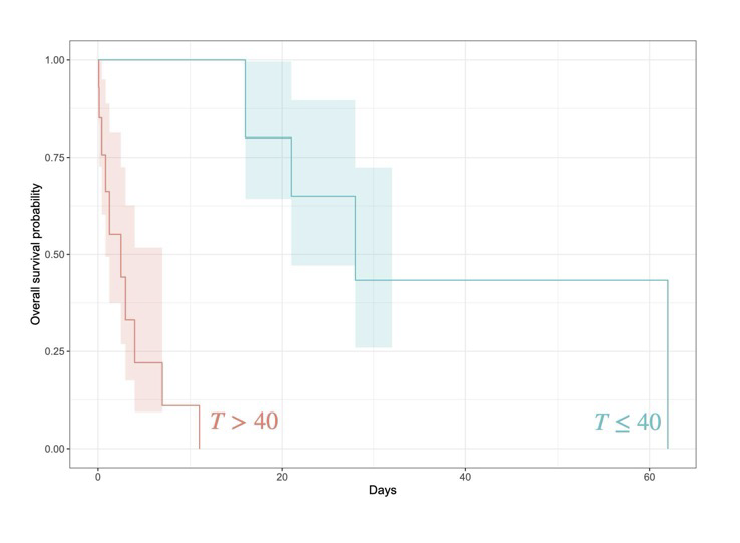

Supplement: S1 Fig — [10] evaluated success or failure of experimental infection of mice following oocyst exposure to different temperatures (35, 40, 45, 50 and 55 degrees for variable durations; n = 62, with 35 events. We digitized the data, and used survival analysis to explore the effects of temperature on oocyst survival. Results from this Kaplan Meier analysis estimates are depicted here and indicate that above 40 degrees celsius, oocyst survival (measured as ability to infect susceptible mice) is considerably reduced. The data and code are available from https://github.com/fidyras/project_toxo. (TIFF) [file pntd.0014058.s001.tiff]

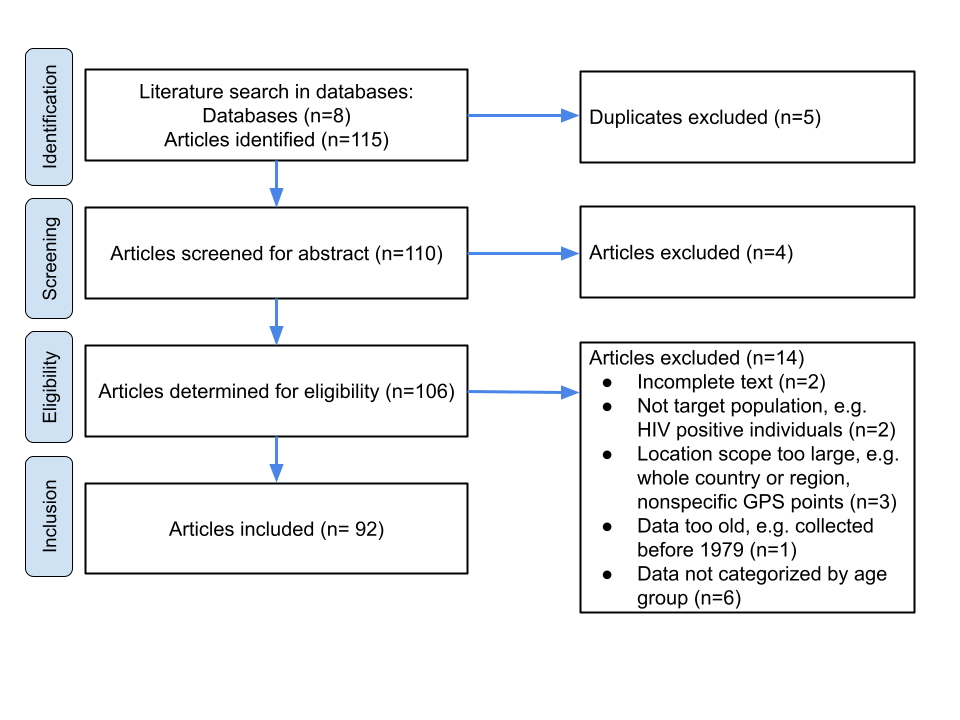

Supplement: S2 Fig — This study was conducted following the 2020 Preferred Reporting Items for Systematic reviews and Meta-Analyses (PRISMA) criteria. Initial literature search was conducted in eight databases using keywords in titles, e.g., “toxoplasmosis seroprevalence africa”. Collected papers were recorded in an excel spreadsheet, as well as saved in PDF form in a OneDrive folder. Duplicate papers were merged. Secondary screenings of abstracts were applied based on inclusion and exclusion criteria, and four papers were excluded due to study not meeting inclusion criteria. The remaining papers were reviewed to see if they matched eligibility criteria. Among excluded criteria, two texts were found to be incomplete, two were with non target populations (e.g., HIV positive individuals), three included a location scope that was too large (e.g., a whole country or region’s data, nonspecific GPS points to view surface temperature), one study was too old (e.g., collected before ERA data began in 1979), and six studies were not categorized by age group and thus ineligible for inclusion. Finally, remaining eligible papers were included and recorded for data analysis in an excel spreadsheet. In search of more papers, the snowball method was used on eligible papers. This method involved screening article titles in the reference section of eligible papers for key phrases such as “toxoplasmosis seroprevalence”. Adding papers by this method allows us to diversify the collection of papers, including to more countries and larger temporal breadth. Articles collected this way underwent the same rigorous screening and are included in the numbers reflected above. In total, 92 papers were determined eligible for study analysis, 72 of which were studies done on pregnant women and women of reproductive age. (TIFF) [file pntd.0014058.s002.tiff]

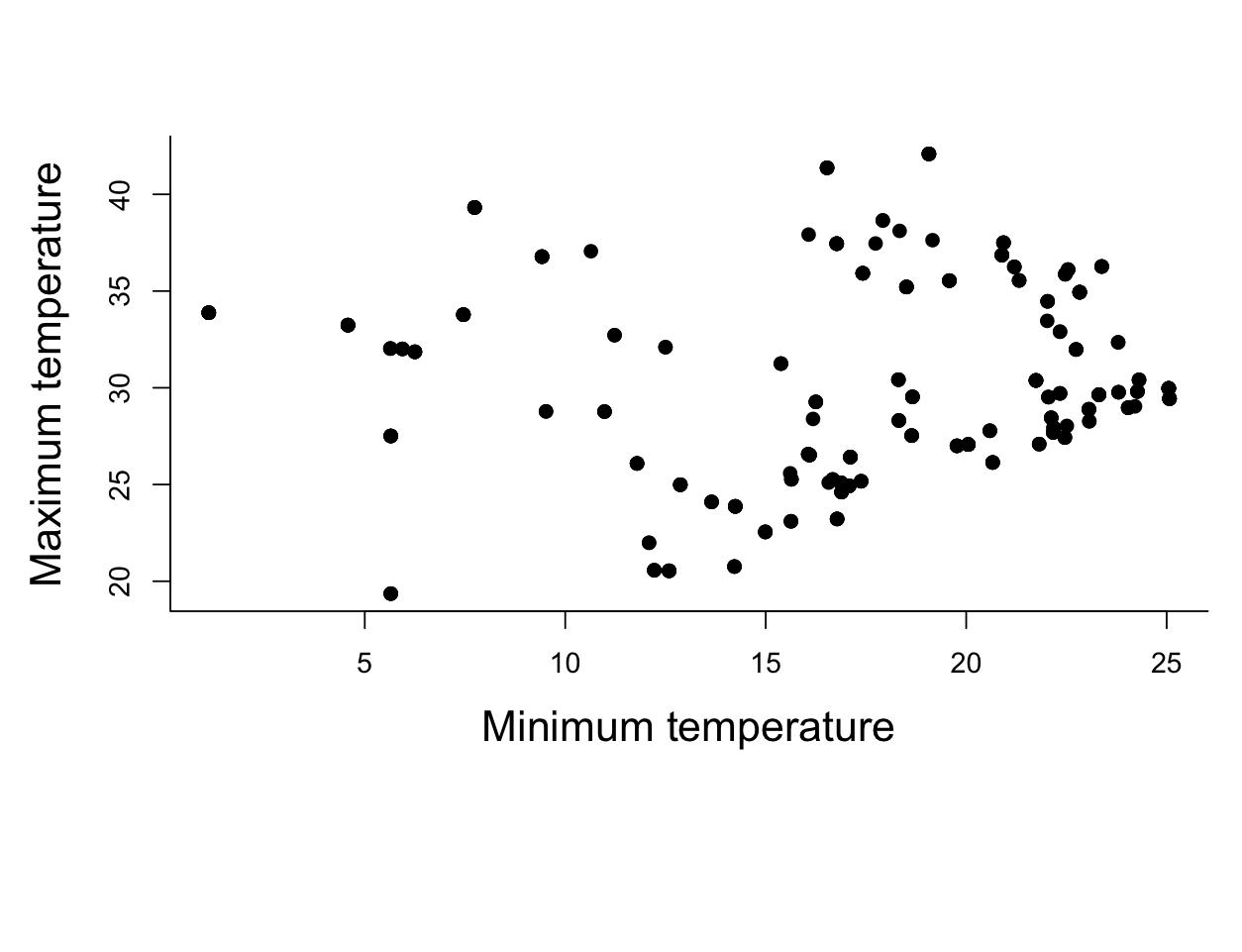

Supplement: S3 Fig — See data for details. (TIFF) [file pntd.0014058.s003.tiff]

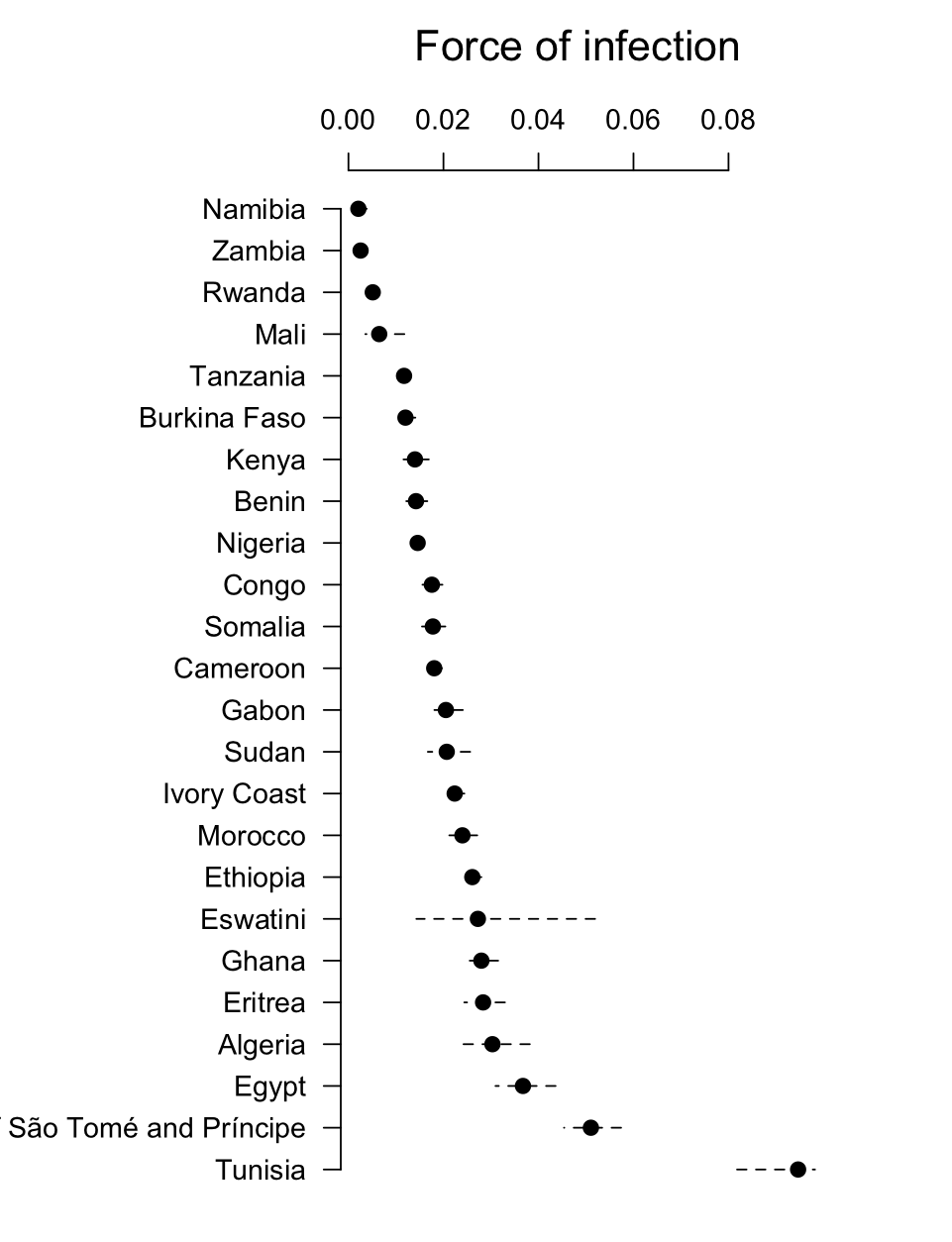

Supplement: S4 Fig — Country level effects from the fitted statistical model depicted in Fig 4, capturing residual variation associated with country to country variation. See S2 Table for the full set of parameter estimates. (TIFF) [file pntd.0014058.s004.tiff]

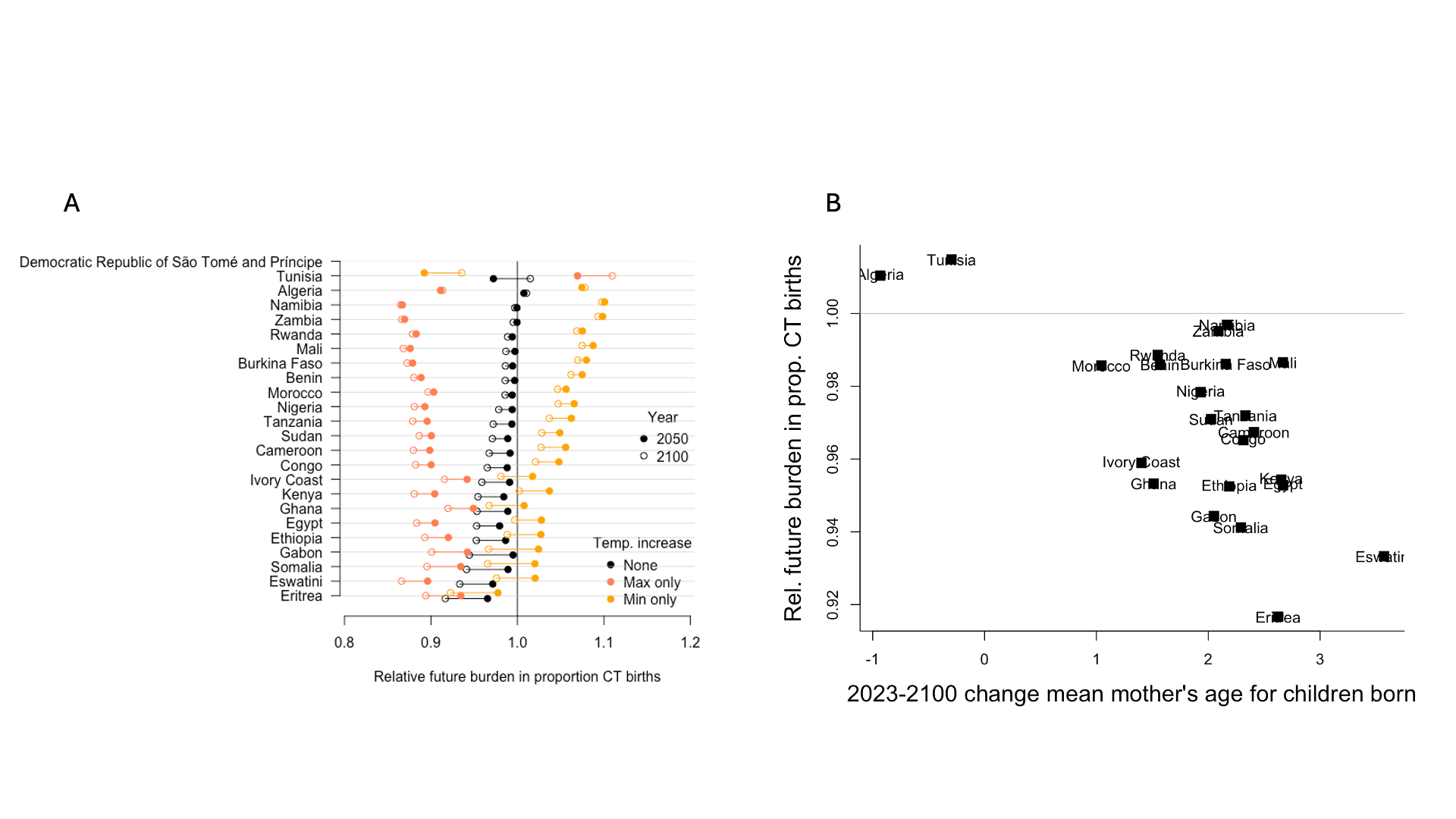

Supplement: S6 Fig — The right hand panel (S6B Fig) indicates the relative future burden in proportion of CT births in 2100 compared to 2023 against the change in mean mother’s age for children born calculated by subtracting values for 2023 from 2100, indicating that as the age at fertility increases (a function of both trends in choice and a shifting population structure) the proportional burden of CT will decline, since most mothers are then likely to be infected prior to pregnancy. Effects of temperature emerge as larger where changes in age of fertility are smallest. (TIFF) [file pntd.0014058.s006.tiff]
