# Supplementary material for: Evaluating how demography and temperature increase might alter the burden of congenital Toxoplasmosis in Africa
Source: PLoS Negl Trop Dis. 2026 Mar 6;20(3):e0014058. doi: 10.1371/journal.pntd.0014058 (PMC12974952; doi:10.1371/journal.pntd.0014058)
Supplement: S5 Fig — Projected relative burden of Toxoplasma gondii infection under scenarios of no temperature change and increases of +1°C, + 2°C, and +3°C in maximum (x-axis) and minimum (y-axis) temperatures for the years 2023, 2050, and 2100. Each panel represents a temperature-change scenario, and projections incorporate corresponding demographic changes for each time period. Colour scales are standardized across panels, with deeper red indicating a higher relative burden and lighter colours indicating a lower relative burden. Future demography has larger effects in countries with ongoing population growth. (PDF) [file pntd.0014058.s005.pdf]

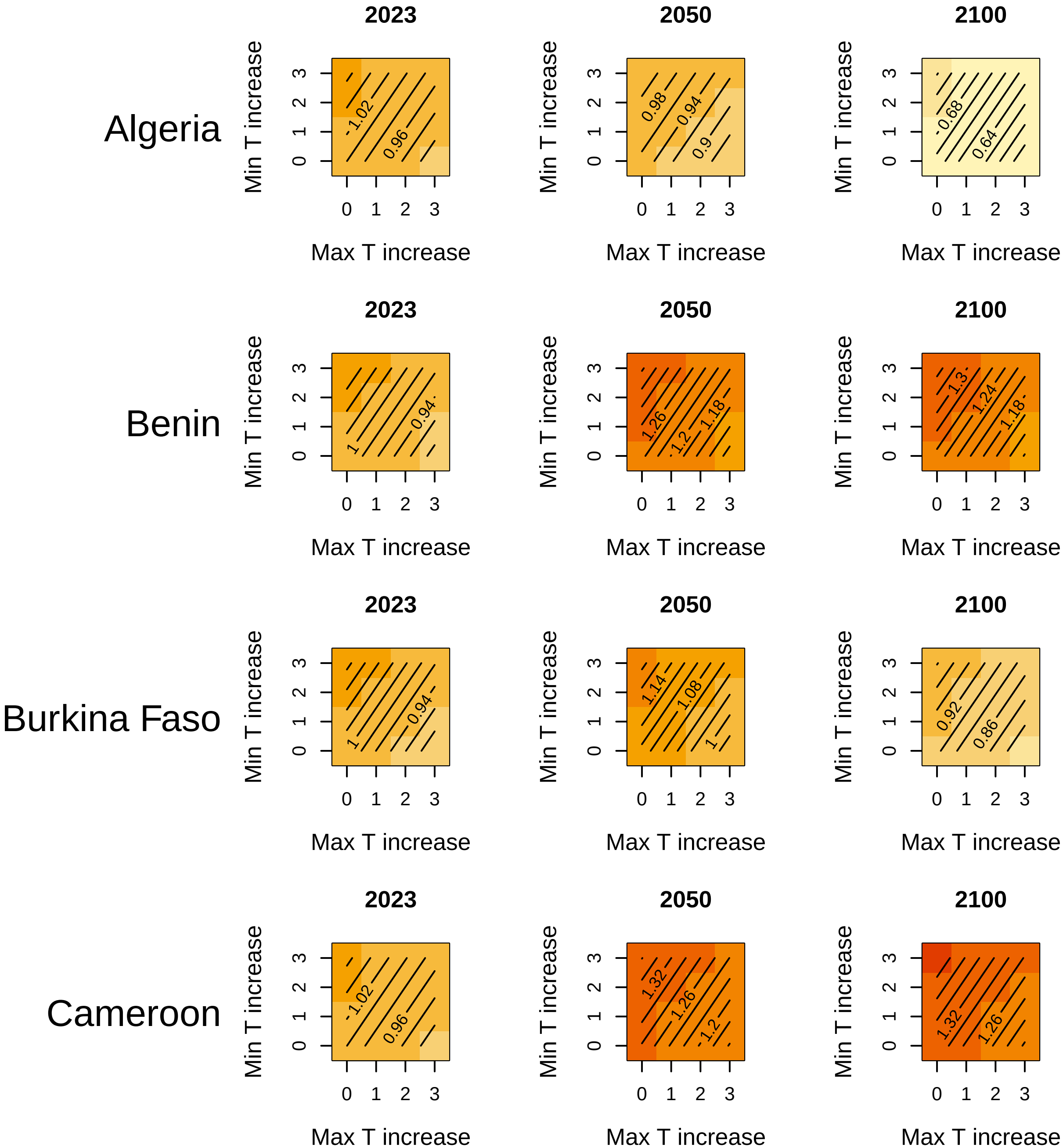
Algeria
Benin
Burkina Faso
Cameroon
2023
2050
2100
Min T increase
Max T increase
0
1
2
3
1.02
0.96
0.98
0.94
0.9
0.68
0.64
1
0.94
1.26
1.2
1.18
1.3
1.24
1.18
1
0.94
1.14
1.08
1
0.92
0.86
1.02
0.96
1.32
1.26
1.2
1.32
1.26

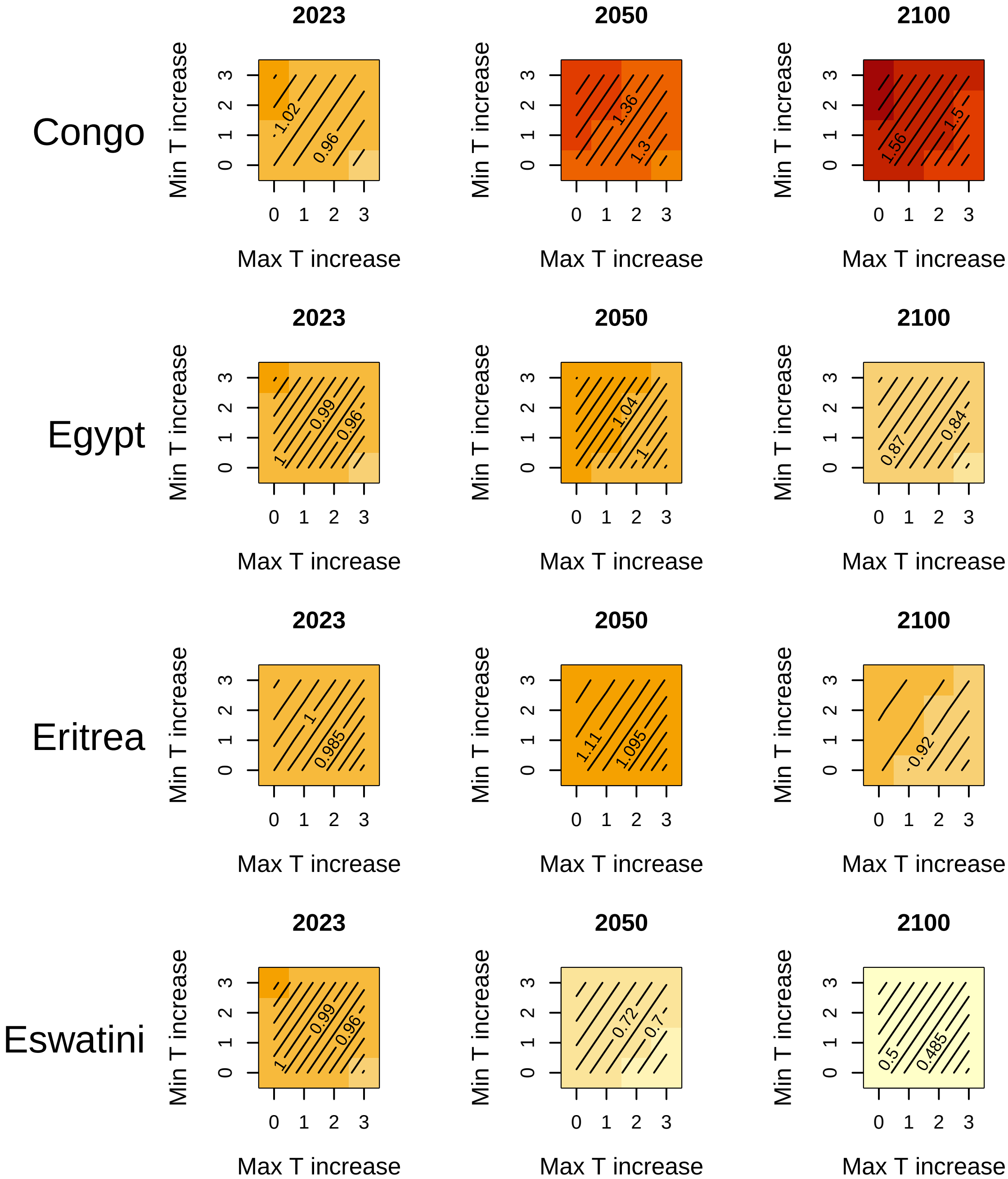

Congo
Egypt
Eritrea
Eswatini
2023
2050
2100
Min T increase
Max T increase
0 1 2 3
1.02
0.96
1.36
1.3
1.56
1.5
0.99
0.96
1
1.04
1
0.87
0.84
1
0.985
1.11
1.095
0.92
0.99
0.96
1
0.72
0.7
0.5
0.485

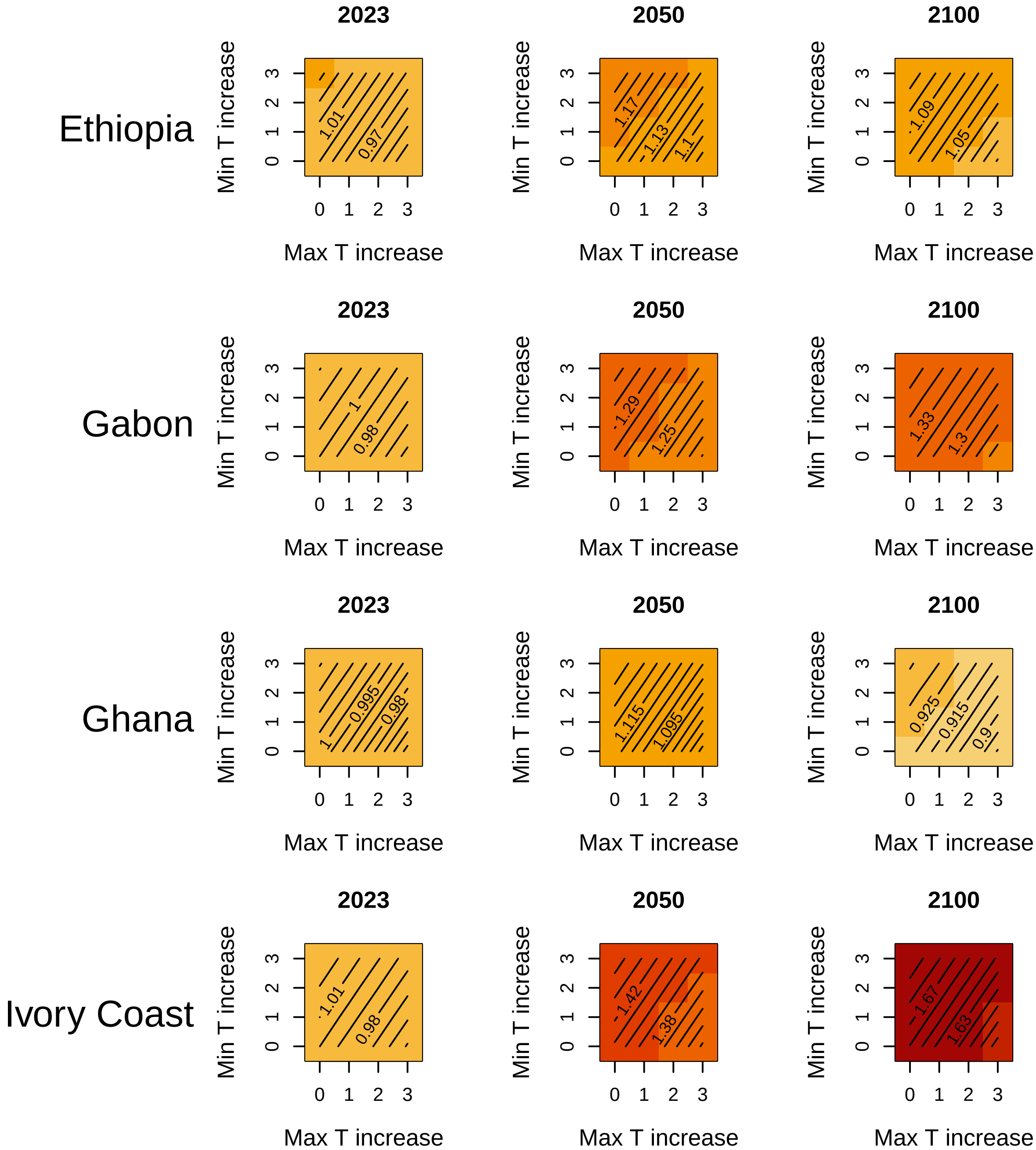
Ethiopia
Gabon
Ghana
Ivory Coast
2023
2050
2100
Min T increase
Max T increase
0
1
2
3
1.01
0.97
1.17
1.13
1.1
1.09
1.05
1
0.98
1.29
1.25
1.33
1.3
0.995
0.98
1
1.115
1.095
0.925
0.915
0.9
1.01
0.98
1.42
1.38
1.67
1.63

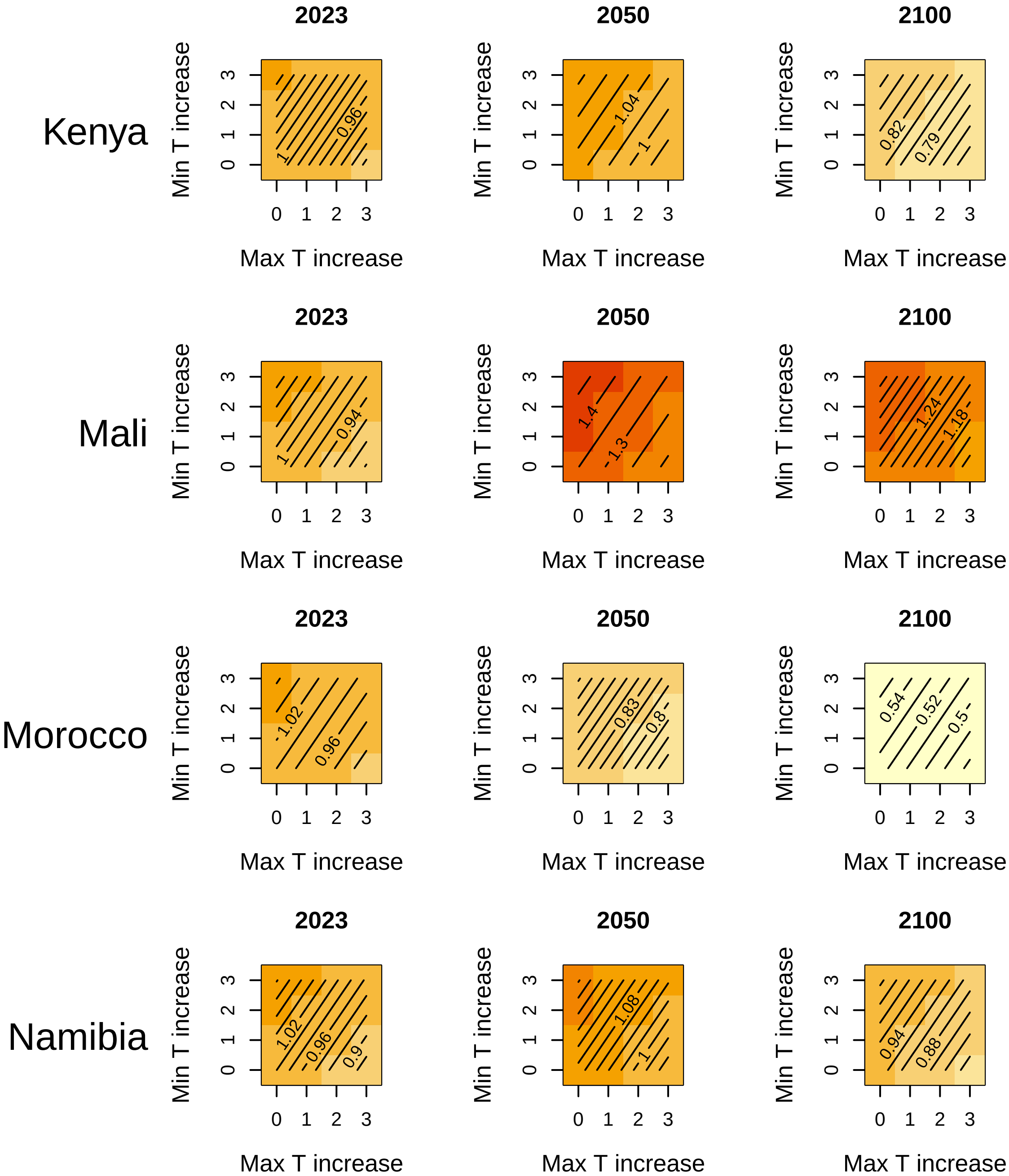
Kenya
Mali
Morocco
Namibia
2023
2050
2100
Min T increase
Max T increase
0
1
2
3
1
0.96
1.04
1
0.82
0.79
1
0.94
1.4
1.3
1.24
1.18
1.02
0.96
0.83
0.8
0.54
0.52
0.5
1.02
0.96
0.9
1.08
1
0.94
0.88

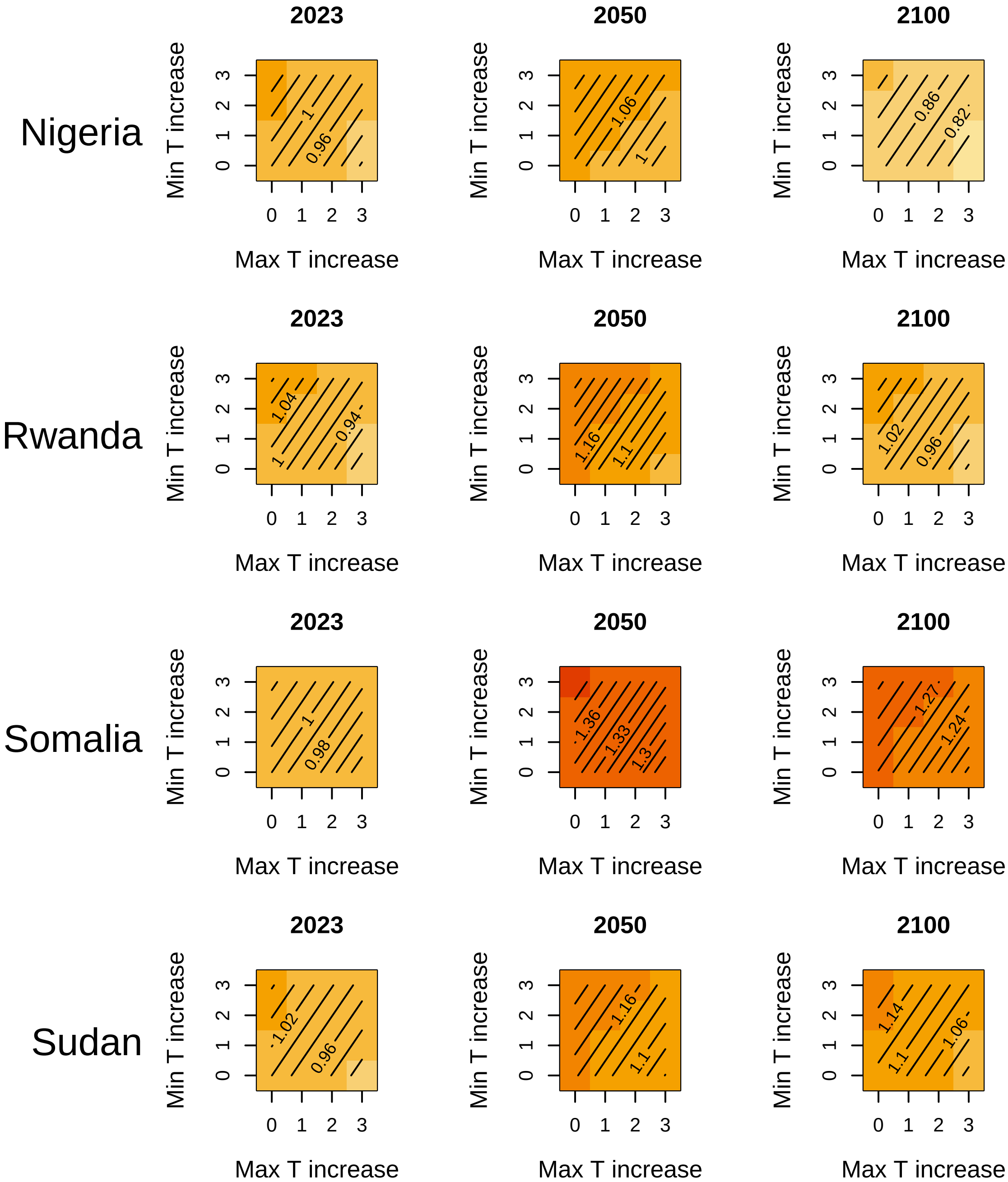
Nigeria
Rwanda
Somalia
Sudan
2023
2050
2100
Min T increase
Max T increase
0 1 2 3
1
0.96
1.06
0.86
0.82
1.04
0.94
1.16
1.1
1.02
0.98
1.36
1.33
1.3
1.27
1.24
1.14

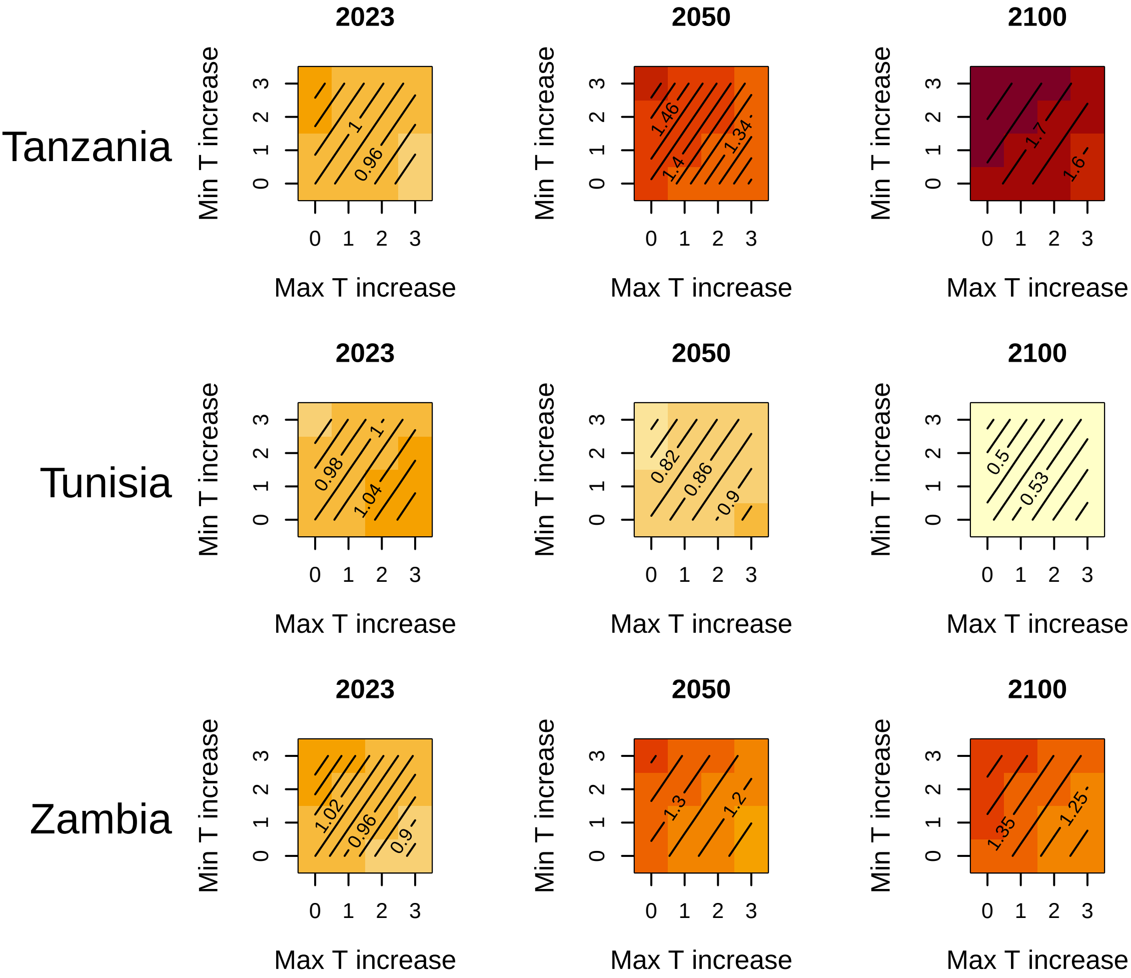
Tanzania
Tunisia
Zambia
2023
2050
2100
Min T increase
Max T increase
0 1 2 3
1
0.96
1.46
1.4
1.34
1.7
1.6
0.98
1-
1.04
0.82
0.86
0.9
0.5
0.53
1.02
0.96
0.9
1.3
1.2
1.35
1.25
