# Supplementary material for: Evaluating how demography and temperature increase might alter the burden of congenital Toxoplasmosis in Africa
Source: PLoS Negl Trop Dis. 2026 Mar 6;20(3):e0014058. doi: 10.1371/journal.pntd.0014058 (PMC12974952; doi:10.1371/journal.pntd.0014058)
Supplement: S1 Table — included in the review. (DOCX) [file pntd.0014058.s007.docx]

S1 Table: Summary of the seroprevalence studies of Toxoplasmosis in pregnant women across Africa included in the review.

| **Authors** | **Country** | **Year** | **Total Positive** | **Total Tested** | **Reference** |
| --- | --- | --- | --- | --- | --- |
| Khames M et al. | Algeria | 2017 | 252 | 1012 | Khames M et al., 2020, Annals of Parasitology |
| Rodier et al. | Benin | 1993 | 113 | 211 | Rodier et al., 1995, Acta Tropica |
| De Paschale et al. | Benin | 2011 | 85 | 283 | De Paschale et al., 2014, Tropical Medicine and International Health |
| Simpore et al. | Burkina Faso | 2004 | 85 | 336 | Simpore et al., 2006, Journal of Medical Virology |
| Bamba S et al. | Burkina Faso | 2012 | 121 | 348 | Bamba S et al., 2014, Pakistan Journal of Biological Sciences |
| Bamba et al. | Burkina Faso | 2013 | 86 | 267 | Bamba et al., 2017, BMC infectious diseases |
| Excler | Burundi | 1985 | 219 | 544 | Excler, 1988, Tropical medicine and parasitology |
| Njunda AL et al. | Cameroon | 2008 | 72 | 110 | Njunda AL et al., 2011, International Journal of Health Research |
| Njunda et al. | Cameroon | 2009 | 77 | 110 | Njunda et al., 2011, J Public Health Africa |
| Assob et al. | Cameroon | 2010 | 93 | 133 | Assob et al., 2011, African Journal of Clinical and Experimental Microbiology |
| Mabeku LB et al. | Cameroon | 2014 | 229 | 643 | Mabeku LB et al., 2018, Journal of Scientific Research and Reports |
| Todjom FG et al. | Cameroon | 2016 | 81 | 176 | Todjom FG et al., 2019, African Journal of Clinical and Experimental Microbiology |
| Abongwa et al. | Cameroon | 2017 | 139 | 606 | Abongwa et al., 2019, Journal of Advances in Microbiology |
| Nguemaim NF et al. | Cameroon | 2018 | 44 | 127 | Nguemaim NF et al., 2020, African Journal of Clinical and Experimental Microbiology |
| Ayeah et al. | Cameroon | 2019 | 236 | 300 | Ayeah et al., 2022, Journal of Parasitology Research |
| Dumas | Congo | 1987 | 701 | 1748 | Dumas, 1990, Annales de la Société belge de médecine tropicale |
| Doudou et al. | Congo | 2011 | 627 | 781 | Doudou et al., 2014, Asian Pacific journal of tropical biomedicine |
| Fan et al. | Democratic Republic of São Tomé and Príncipe | 2003 | 26 | 121 | Fan et al., 2006, Transactions of The Royal Society of Tropical Medicine and Hygiene |
| Hung et al. | Democratic Republic of São Tomé and Príncipe | 2003 | 321 | 436 | Hung et al., 2007, Transactions of The Royal Society of Tropical Medicine and Hygiene |
| Fan et al. | Democratic Republic of São Tomé and Príncipe | 2010 | 131 | 196 | Fan et al., 2012, Parasites & vectors |
| El Deeb et al. | Egypt | 2010 | 218 | 323 | El Deeb et al., 2012, Acta Tropica |
| Hafez Hassanain et al. | Egypt | 2016 | 79 | 388 | Hafez Hassanain et al., 2018, Pakistan Journal of Biological Sciences: PJBS |
| Abdelbaset AE et al. | Egypt | 2017 | 20 | 88 | Abdelbaset AE et al., 2020, Veterinary World |
| Tecle AS et al. | Eritrea | 2016 | 112 | 210 | Tecle AS et al., 2020, International Journal of Medical Parisitology & Epidemiology Sciences |
| Lio et al | Eswatini | 2009 | 9 | 93 | Lio et al, 2009, Annals of Tropical Medicine & Parasitology |
| Negash et al. | Ethiopia | 2007 | 40 | 68 | Negash et al., 2007, Central African journal of medicine |
| Shimelis et al. | Ethiopia | 2007 | 209 | 238 | Shimelis et al., 2009, BMC Res Notes |
| Endris M et al. | Ethiopia | 2010 | 341 | 385 | Endris M et al., 2014, Iranian journal of parasitology |
| Gelaye et al. | Ethiopia | 2010 | 246 | 288 | Gelaye et al., 2015, International Journal of Infectious Diseases |
| Zemene et al. | Ethiopia | 2011 | 168 | 201 | Zemene et al., 2012, BMC infectious diseases |
| Agmas et al. | Ethiopia | 2013 | 180 | 263 | Agmas et al., 2015, BMC research notes |
| Awoke K et al. | Ethiopia | 2013 | 71 | 384 | Awoke K et al., 2015, Asian Pacific journal of tropical medicine |
| Negussie A et al. | Ethiopia | 2013 | 105 | 301 | Negussie A et al., 2017, Int J Trop Dis Health |
| Walle et al. | Ethiopia | 2013 | 71 | 100 | Walle et al., 2013, Parasites & Vectors |
| Jula et al. | Ethiopia | 2015 | 96 | 396 | Jula et al., 2018, Revista Española de Quimioterapia |
| Mulugeta et al. | Ethiopia | 2015 | 148 | 233 | Mulugeta et al., 2020, Infectious Diseases: Research and Treatment 13 |
| Yohanes T et al. | Ethiopia | 2015 | 184 | 232 | Yohanes T et al., 2017, Transl Biomed |
| Fenta | Ethiopia | 2016 | 382 | 494 | Fenta., 2019, BMC infectious diseases |
| Negero et al. | Ethiopia | 2016 | 159 | 210 | Negero et al., 2017, International Journal of Infectious Diseases |
| Teweldmedihin et al. | Ethiopia | 2018 | 128 | 360 | Teweldmedihin et al., 2019, BMC infectious diseases |
| Adunga B et al. | Ethiopia | 2019 | 165 | 401 | Adunga B et al., 2021, Infection and Drug Resistanc |
| Guebre-Xavier | Ethiopia* | 1990 | 756 | 1016 | Guebre-Xavier, 1993, Ethiopian medical journal |
| Pegha Moukandija et al. | Gabon | 2007 | 416 | 973 | Pegha Moukandija et al., 2017, BMC Pregnancy and Childbirth |
| Ayi I et al. | Ghana | 2015 | 64 | 125 | Ayi I et al., 2016, Tropical medicine and health |
| Kwofie KD et al. | Ghana | 2015 | 35 | 93 | Kwofie KD et al., 2016, Maternal and child health journal |
| Singh B et al. | Ghana | 2017 | 221 | 385 | Singh B et al., 2021, Infectious Diseases in Obstetrics and Gynecology |
| Volker et al. | Ghana | 2017 | 123 | 168 | Volker et al., 2017, BMC pregnancy and childbirth |
| Agordzo et al. | Ghana | 2018 | 111 | 242 | Agordzo et al., 2020, AAS Open Res |
| Adou-Bryn et al. | Ivory Coast | 2000 | 579 | 1025 | Adou-Bryn et al., 2004, Bulletin de la Société de Pathologie Exotique |
| Koffi et al. | Ivory Coast | 2014 | 226 | 385 | Koffi et al., 2015, International Journal of Tropical Disease and Health |
| Hunsperger et al. | Kenya | 2017 | 109 | 351 | Hunsperger et al., 2024, Epidemiology and infection |
| Ouologuem et al. | Mali | 2007 | 21 | 288 | Ouologuem et al., 2013, The Journal of parasitology |
| Tlamcani Z et al. | Morocco | 2010 | 284 | 1367 | Tlamcani Z et al., 2017, Acta Medica International |
| Hoummadi L et al. | Morocco | 2014 | 1490 | 5078 | Hoummadi L et al., 2020, African health sciences |
| Laboudi M et al. | Morocco | 2014 | 248 | 576 | Laboudi M et al., 2021, Tropical Medicine and Health |
| Van der Colf BE et al. | Namibia | 2016 | 9 | 344 | Van der Colf BE et al., 2020, Southern African Journal of Infectious Diseases |
| Olusi et al. | Nigeria | 1995 | 259 | 606 | Olusi et al., 1996, Scandanavian Journal of Infectious Diseases |
| Uneke et al. | Nigeria | 2007 | 30 | 134 | Uneke et al., 2007, African journal of medicine and medical sciences |
| Kamani et al. | Nigeria | 2008 | 42 | 170 | Kamani et al., 2009, Annals of Tropical Medicine & Parasitology |
| Osunkalu et al. | Nigeria | 2010 | 180 | 308 | Osunkalu et al., 2011, HIV/AIDS - Research and Palliative Care |
| Alayande et al. | Nigeria | 2011 | 22 | 84 | Alayande et al., 2012, African Journal of Microbiology Research |
| Gyang et al. | Nigeria | 2013 | 52 | 226 | Gyang et al., 2015, Revista da Sociedade Brasileira de Medicina Tropica |
| Oboro IL et al. | Nigeria | 2013 | 189 | 288 | Oboro IL et al., 2016, Nigerian Health Journal |
| Oyinloye SO et al. | Nigeria | 2013 | 19 | 87 | Oyinloye SO et al., 2014, African Journal of Clinical and Experimental Microbiology |
| Nasir IA et al. | Nigeria | 2014 | 100 | 360 | Nasir IA et al., 2015, Journal of Medical Sciences |
| Yusuf et al. | Nigeria | 2015 | 130 | 200 | Yusuf et al., 2017, Borno Med Journal |
| Ballah F et al. | Nigeria | 2016 | 112 | 400 | Ballah F et al., 2017, Asian Journal of Medicine and Health |
| Ibrahim et al. | Nigeria | 2016 | 24 | 320 | Ibrahim et al., 2017, UJMR |
| Sowemimo et al. | Nigeria | 2016 | 19 | 272 | Sowemimo et al., 2018, Trans R Soc Trop Med Hyg |
| Woken et al | Nigeria | 2016 | 56 | 206 | Woken et al,. 2018, Nigerian Journal of Parisitology |
| Adeniyi OT et al. | Nigeria | 2017 | 181 | 369 | Adeniyi OT et al., 2018, Journal of Infectious Diseases and Immunity |
| Zakari M et al. | Nigeria | 2017 | 47 | 155 | Zakari M et al., 2020, Malawi Medical Journa |
| Blackburn et al. | Nigeria | 2018 | 12148 | 44269 | Blackburn et al., 2024, The American journal of tropical medicine and hygiene |
| Akubuilo AS et al. | Nigeria | 2019 | 45 | 384 | Akubuilo AS et al., 2020, Open Journal of Bioscience Research |
| Okojokwo O et al. | Nigeria | 2020 | 42 | 158 | Okojokwo O et al., 2023, Microbes and Infectious Diseases |
| David et al | Nigeria | 2023 | 27 | 104 | David et al, 2023, Microbes and Infectious Diseases |
| Gascon et al. | Rwanda | 1989 | 32 | 272 | Gascon et al., 1989, Revista do Instituto de Medicina Tropical de São Paulo |
| Murebwayire E et al. | Rwanda | 2014 | 47 | 384 | Murebwayire E et al., 2017, Tanzania Journal of Health Research |
| Ahmed et al. | Somalia | 1987 | 236 | 566 | Ahmed et al., 1988, Transactions of the Royal Society of Tropical Medicine and Hygiene |
| Hassan SA et al. | Somalia | 2021 | 182 | 403 | Hassan SA et al., 2023, Acta Tropica |
| Abdel-Hameed | Sudan | 1991 | 69 | 195 | Abdel-Hameed, 1991, Journal of Tropical Medicine and Journal of ropical medicine and hygiene |
| Satti A.B et al. | Sudan | 2006 | 53 | 234 | Satti A.B et al., 2011, Sudan Medical Laboratory Journal |
| Abdel-Raouff M et al. | Sudan | 2013 | 130 | 163 | Abdel-Raouff M et al., 2014, Journal of Coastal Life Medicine |
| Swai et al. | Tanzania | 2005 | 90 | 199 | Swai et al., 2009, Tanzana Journal of Health Research |
| Mwambe et al. | Tanzania | 2012 | 109 | 350 | Mwambe et al., 2013, Parasites & vectors |
| Saajan AM et al. | Tanzania | 2013 | 123 | 310 | Saajan AM et al., 2017, The East African Health Research Journal |
| Shao et al. | Tanzania | 2014 | 60 | 144 | Shao et al., 2015, Annals of Clinical and Laboratory Research |
| Mirambo MM et al | Tanzania | 2015 | 92 | 300 | Mirambo MM et al., 2019, African Health Sciences |
| Paul et al. | Tanzania | 2017 | 92 | 214 | Paul et al., 2018, Tropical medicine and health |
| Lushina M et al. | Tanzania | 2020 | 59 | 172 | Lushina M et al., 2022, East African Health Research Journal |
| Bouratbine et al. | Tunisia | 2001 | 405 | 828 | Bouratbine et al., 2001, Parasite |
| Frimpong et al. | Zambia | 2015 | 24 | 411 | Frimpong et al., 2017, BMC infectious diseases |
