# Supplementary material for: Evaluating how demography and temperature increase might alter the burden of congenital Toxoplasmosis in Africa
Source: PLoS Negl Trop Dis. 2026 Mar 6;20(3):e0014058. doi: 10.1371/journal.pntd.0014058 (PMC12974952; doi:10.1371/journal.pntd.0014058)
Supplement: S2 Table — (DOCX) [file pntd.0014058.s008.docx]

| Country | Estimate | Standard Error |
| --- | --- | --- |
| Algeria | -3.275 | 0.122 |
| Benin | -4.378 | 0.079 |
| Burkina Faso | -4.615 | 0.077 |
| Cameroon | -4.085 | 0.043 |
| Congo | -3.830 | 0.051 |
| Democratic Republic of São Tomé and Príncipe | -3.147 | 0.060 |
| Egypt | -3.217 | 0.087 |
| Eritrea | -3.590 | 0.077 |
| Eswatini | -3.529 | 0.330 |
| Ethiopia | -3.697 | 0.034 |
| Gabon | -4.026 | 0.078 |
| Ghana | -3.743 | 0.058 |
| Côte d’Ivoire (Ivory Coast) | -3.935 | 0.046 |
| Kenya | -4.419 | 0.094 |
| Mali | -5.102 | 0.303 |
| Mauritania | -4.043 | 0.043 |
| Morocco | -3.607 | 0.066 |
| Namibia | -6.040 | 0.313 |
| Nigeria | -4.278 | 0.029 |
| Rwanda | -5.293 | 0.116 |
| Somalia | -4.176 | 0.071 |
| Sudan | -3.496 | 0.080 |
| Tanzania | -4.543 | 0.044 |
| Tunisia | -2.211 | 0.088 |
| Zambia | -5.979 | 0.199 |
