# Supplementary material for: Evaluating how demography and temperature increase might alter the burden of congenital Toxoplasmosis in Africa
Source: PLoS Negl Trop Dis. 2026 Mar 6;20(3):e0014058. doi: 10.1371/journal.pntd.0014058 (PMC12974952; doi:10.1371/journal.pntd.0014058)
Supplement: S3 Table — Projected burden was obtained by estimating the force of infection for each country assuming that HDI was unchanged, but maximum and minimum temperature increased by 0–3 degrees celsius (resulting in 16 possible combinations of minimum and maximum). The resulting age profile of first infection was combined with projected number of children born to women of age a in the focal year (2023, 2050 and 2100). Relative burden was obtained by standardizing by dividing each value by estimates obtained for that country in 2023 with no temperature change. We fitted minimum and maximum temperature as quantitative variables, alongside year and HDI, and an interaction between the latter two (r2 = 0.25). (DOCX) [file pntd.0014058.s009.docx]

**S3 Table: Parameters for a model synthesizing effects on log projected relative burden.**

| **Parameter** | **Estimate** | **Standard Error** | **P-value** |
| --- | --- | --- | --- |
| Intercept (no temperature change) | 78.380 | 6.747 | <0.001 |
| Change of max temperature | -0.029 | 0.005 | <0.001 |
| Change of minimum temperature | 0.028 | 0.005 | <0.001 |
| Year | -0.039 | 0.003 | <0.001 |
| HDI | -140.5 | 12.11 | <0.001 |
| Year x HDI | 0.070 | 0.005 | <0.001 |
