# Supplementary material for: Evaluating how demography and temperature increase might alter the burden of congenital Toxoplasmosis in Africa
Source: PLoS Negl Trop Dis. 2026 Mar 6;20(3):e0014058. doi: 10.1371/journal.pntd.0014058 (PMC12974952; doi:10.1371/journal.pntd.0014058)
Supplement: Attachment — Submitted filename: Response to reviewers_1_202601.docx [file pntd.0014058.s010.docx]

Fidisoa Rasambainarivo
on behalf of all co-authors

Editors of
PLOS Neglected Tropical Diseases

Many thanks for the invitation to resubmit our manuscript. Below, we provide a response to each of the reviewer suggestions in blue.

**Additional Editor Comments :**

I agree with reviewer 2's comment that the figures are generally difficult to read and understand. Please revise them to make them easier to see.

To try and improve this, we revisited the figures. We altered the structure and caption of Figure 1 and the caption and labelling of Figure 2. For Figure 3, we increased the spacing and font size and removed the temperature ranges to the supplement; we also removed the exemplar fertility curve for Figure 3 altogether as a range of examples are provided in Figure 5. For Figure 4, we increased spacing and font size, made the y axis consistent in range across the three panels, and also moved the plot of the random effects to the supplement (new Figure S4). For Figure 5, we made the core panel (projected burden) much bigger, removed one of the examples as it proved to have little explanatory power (changing both min and max temperature together) and positioned the exemplar fertility curves near their location on the plot, removed the overlaid FOI curves. We think this much better conveys the fact that countries expecting a larger delay in fertility are much more likely to see increases in burden, and have also tried to clarify this in the text. We also explored separating out onto different plots all the different sources of burden - however, this makes comparison much less straightforward, so we chose to retain the current framing, but are very happy to revisit.

I agree with reviewer 3, who pointed out that one limitation of this study is that it assumed a temperature rise of 3 degrees. It would be even better to perform a sensitivity test to see how sensitive the results are to temperature changes (e.g., 1, 2, and 3 degrees change).

Many thanks for this useful suggestion - we now include the range of different temperatures in the supplement, specifically no increase, and a 1, 2 and 3 degree increase to both minimum and maximum temperatures in the supplement (Figure S5). To interpret this set of results (which translates to 1,200 new datapoints), we used the regression approach presented in the previous version of the manuscript, but now using the quantitative values of minimum and maximum temperature rather than ‘the generic ‘increase’ and ‘decrease’. We have updated the results to reflect this.

We also now include in the supplement the full set of figures indicating the relative burden across different time horizons for increases in the maximum and minimum temperature (Figure S5).

I failed to understand the mechanism by which rising minimum temperatures increases the force of infection (Figure 4C). Please explain the hypothesized mechanism behind.

We have worked to better introduce this theme in the introduction, expanding the third paragraph so that it now reads:

“Age seroprevalence studies can be used to estimate the force of infection, revealing patterns of risk across different settings [[9]](https://paperpile.com/c/eFk1Tx/XP3x). Age seroprevalence for *T. gondii* in human populations is likely to be context specific, as the life cycle of this protozoan pathogen is mediated by behavioral and social features, such as food preparation methods, but also the environment (Figure 1). Humans and other vertebrates can be infected with T. gondii via two different paths: i) ingestion of T. gondii cysts by consumption of an infected animal; and ii) ingestion of oocysts directly from the environment (e.g., via unwashed leafy vegetables). Oocysts shed in the feces of cats are an important source of infection for vertebrates including humans. Crucially, experimental evidence indicates that the survival of *T. gondii* oocysts in the environment is reduced at high temperatures [[10]](https://paperpile.com/c/eFk1Tx/nxEKB). Although processes in natural systems are unlikely to map perfectly to tightly controlled experiments, this experimental result, alongside evidence that oocyst survival is reduced on freezing (Kuticic and Wikerhauser 1996) suggests that oocysts are sensitive to temperature, and thus might have performance limits at higher and lower levels of temperature that could modulate overall transmission of the pathogen. This latter point was our focus in this paper: the age specific manifestation of the main burden of this infection in humans means that the effects of climate change on oocyst survival might result in large shifts in the impact on population health by reducing environmental exposure (Figure 1, red arrows) by diminishing survival of oocysts, which will reduce the rate at which susceptible individuals become infected, or the force of infection (Figure 2). However, other dependencies including on local demography mean that the mapping is unlikely to be straightforward. ”

As indicated in the text, we also now flag the core transition expected to be affected by a change in temperature (red arrows, with explanatory text) on Figure 1. We have also tried to better detail the source of our expectation that temperature will affect this transition.

There may be collinearity between the minimum and maximum temperatures, which are incorporated as independent variables into the single statistical model (pages 9-10). Collinearity might make the regression model unstable, so it is recommended that the authors test for collinearity using VIF or Pearson r.

We tested for collinearity using the R package *rvif* and now reported this in the methods section:

“Since biological limits across abiotic variables like temperature are expected, we included both annual minimum and maximum of the daily mean temperatures. The variance inflation factor (calculated using the R package rvif) indicated that multicollinearity between these two variables was sufficiently small as to not destabilize the regression (V=1.08) as indicated in Figure S3. ”

Related to the question above, I failed to understand why the model includes two very similar, potentially interrelated explanatory variables (i.e., maximum and minimum temperatures) that might be causing the collinearity issue. Wouldn't it be more reasonable to use either the average temperature, the maximum temperature, or the minimum temperature and build a separate model for each temperature index (i.e., a total of 3 separate regression models are built with 3 different temperature index) and compare the results across temperatures? Please explain (or refute) why two potentially interrelated variables have to be incorporated into a single regression model.

As indicated above, the correlation between the minimum and maximum temperature is small ($\rho=0.01$, translating to at VIF close to 1). Furthermore, we expect these extremes of temperature both to have distinct effects on the force of infection as each represents a different (and uncorrelated in our data, now depicted in Figure S3) biological limit.

We experimented with using the median temperature as a single summary variable of temperature in each setting. However, the associated regression proved very unstable (both fluctuating across temperature in a biologically unlikely fashion, and sensitive to removal of particular datapoints). As the signal associated with hard biological limits (temperatures above or below which oocysts survival is impossible) appears to be much crisper than that associated with the median temperature (possibly because of the complexity of the life cycle), and the estimates seem biologically reasonable (an increase and a decline) we chose to retain our existing formulation.

The provided Github link cannot be reached, so please check the address of the link.

Thank you, we have updated the Github link and can now be accessed at <https://github.com/fidyras/Project_Toxo>

**Journal Requirements:**

1) Please ensure that the CRediT author contributions listed for every co-author are completed accurately and in full.
At this stage, the following Authors/Authors require contributions: Fidisoa T Rasambainarivo, Ingrid G Nilsson, Devin E Cheeks, Wenchang Yang, and C. Jessica E. Metcalf. Please ensure that the full contributions of each author are acknowledged in the "Add/Edit/Remove Authors" section of our submission form.
The list of CRediT author contributions may be found here: <https://journals.plos.org/plosntds/s/authorship#loc-author-contributions>

Done

2) We ask that a manuscript source file is provided at Revision. Please upload your manuscript file as a .doc, .docx, .rtf or .tex. If you are providing a .tex file, please upload it under the item type u2018LaTeX Source Fileu2019 and leave your .pdf version as the item type u2018Manuscriptu2019.

3) Please provide an Author Summary. This should appear in your manuscript between the Abstract (if applicable) and the Introduction, and should be 150-200 words long. The aim should be to make your findings accessible to a wide audience that includes both scientists and non-scientists. Sample summaries can be found on our website under Submission Guidelines:
<https://journals.plos.org/plosntds/s/submission-guidelines#loc-parts-of-a-submission>

Included as requested:

“The pathogen *Toxoplasma gondii* is associated with serious health outcomes if women are infected for the first time during pregnancy. In Sub-Saharan Africa, transmission is currently sufficiently high that most women are infected before their child-bearing years. However, higher temperatures might reduce survival of the pathogen’s environmental life stage (oocysts). This could reduce transmission and put more women at risk. We evaluate the potential shift in burden associated with a changing climate in the context of changing patterns of fertility over age, identifying settings where an increase might be expected.”

4) Please upload all main figures as separate Figure files in .tif or .eps format. For more information about how to convert and format your figure files please see our guidelines:
<https://journals.plos.org/plosntds/s/figures>

Done

5) We have noticed that you have uploaded Supporting Information files, but you have not included a complete list of legends. Please add a full list of legends for your Supporting Information file (Table S1) after the references list.

Done

6) We notice that your supplementary Figures are included in the manuscript file. Please remove them and upload them with the file type 'Supporting Information'. Please ensure that each Supporting Information file has a legend listed in the manuscript after the references list.

Done

7) Some material included in your submission may be copyrighted. According to PLOSu2019s copyright policy, authors who use figures or other material (e.g., graphics, clipart, maps) from another author or copyright holder must demonstrate or obtain permission to publish this material under the Creative Commons Attribution 4.0 International (CC BY 4.0) License used by PLOS journals. Please closely review the details of PLOSu2019s copyright requirements here: PLOS Licenses and Copyright. If you need to request permissions from a copyright holder, you may use PLOS's Copyright Content Permission form.
Please respond directly to this email and provide any known details concerning your material's license terms and permissions required for reuse, even if you have not yet obtained copyright permissions or are unsure of your material's copyright compatibility. Once you have responded and addressed all other outstanding technical requirements, you may resubmit your manuscript within Editorial Manager.
Potential Copyright Issues:

i) Figure 1. Please confirm whether you drew the images / clip-art within the figure panels by hand. If you did not draw the images, please provide (a) a link to the source of the images or icons and their license / terms of use; or (b) written permission from the copyright holder to publish the images or icons under our CC BY 4.0 license. Alternatively, you may replace the images with open source alternatives. See these open source resources you may use to replace images / clip-art:
- [https://commons.wikimedia.org](https://commons.wikimedia.org/)
- <https://openclipart.org/>.

done with biorender (with written permission/license)

ii) Figure S2 . We noted that you stated in the figure legend "Dubey et al. [10] ." Please clarify whether the figure is obtained from a previously published article. If so, please include a direct link to source of the figure in the legend and a link to the terms of use/license information.

This analysis and the Figure was developed based on the data provided in Dubey et al. We have now updated the Figure Legend to make this clearer (noting also that in this version we have updated the order of supplementary Figures):

“***Figure S1*: Effect of temperature on oocyst survival.** Dubey et al. [[10]](https://paperpile.com/c/eFk1Tx/nxEKB) evaluated success or failure of experimental infection of mice following oocyst exposure to different temperatures (35, 40, 45, 50 and 55 degrees for variable durations; n= 62, with 35 events, see Supplementary R Code). We digitized the data (values provided in the Supplementary R Code), and used survival analysis to explore the effects of temperature on oocyst survival. Results from this Kaplan Meier analysis are depicted here and indicate that above 40 degrees celsius, oocyst survival (measured as ability to infect susceptible mice) is considerably reduced.”

We have also indicated this in the main text on first mention.

“Crucially, experimental evidence indicates that the survival of T. gondii oocysts in the environment is reduced at high temperatures [10] (data from this paper is presented in Figure S1)”

8) Thank you for stating "All data used in this study is open access and available freely on the internet, see the methods section for more details." Please amend your Data Availability Statement to include the full link(s) to the data.

Done

9) You indicated that "Data and code used to produce this analysis is available from a Github repository cited in the manuscripthttps://github.com/fidyras/project_toxo." When we accessed the provided link, this message was displayed "This repository is empty." Please provide us with the link where the data can be found.

Done

10) Please revise your current Competing Interest statement to the standard "The authors have declared that no competing interests exist."

Done

**Reviewers' Comments:**

Reviewer #1: The authors attempt to estimate the future burden of congenital toxoplasmosis in Africa under scenarios of demographic and climate change. The hypothesis that climate change could reduce the force of infection and thereby increase disease burden is interesting. However, I have several concerns about the study design and interpretation.

1) Toxoplasma gondii is transmitted by at least two major routes — ingestion of oocysts and ingestion of tissue cysts — and ingestion of tissue cysts is generally considered the dominant route in many settings. The relative importance of these routes can vary by country and context. The manuscript should explicitly acknowledge both oocyst- and tissue cyst–mediated transmission.

This is an excellent point and apologies for not providing clarity on this in the previous version of the manuscript. We have tried to strength our presentation of this point first by editing the text of the manuscript, now stating in the introduction:

“Humans and other vertebrates can be infected with *T. gondii* via two different paths: i) ingestion of T. gondii cysts by consumption of an infected animal; and ii) ingestion of oocysts directly from the environment (e.g., via unwashed leafy vegetables). ”

And second, by editing Figure 1 to reveal these two different paths.

2) The hypothesis that higher temperatures reduce oocyst survival is derived from experimental studies conducted in warm water baths, which may not reflect the environmental conditions oocysts actually experience in the field under climate change. Although the fitted model shows a negative association between Tmax and the force of infection, the model explains only ~52% of the variation. To strengthen the argument, I recommend visually comparing the observed seroprevalence data with the temperature distributions (e.g., Tmin/Tmax ranges) across countries or study sites so readers can better assess whether the temperature–seroprevalence relationship is consistent and biologically plausible.

We now mention the possibility of imperfect mapping from temperature ranges in experimental systems more clearly at first mention of the Dubey et al. results:

“Although processes in natural systems are unlikely to map perfectly to tightly controlled experiments, this result suggests that oocysts are sensitive to temperature, and thus might have performance limits at higher and lower levels of temperature that could modulate overall transmission of the pathogen.”

Figure 3 represents the full scope of the data and includes a colour range to represent the effects of temperature. The concentration of the warmer colours towards the bottom of the plot broadly indicate the impact of temperature (and we have now expanded the caption of the Figure to indicate this); however, there are of course other sources of variation. The ideal data with which to represent the temperature effect would contain seroprevalence curves from the same location at several points in time corresponding to different temperatures yet the same level of HDI - the expectation would then be that curves corresponding to times of higher temperature would increase much more slowly. Cities for which more than one seroprevalence curve are available unfortunately only show minimal variation in temperature and considerable variation in HDI (e.g., measurements from Addis Ababa).

3) There is not much explanation why Tmin is in positive correlation with the force of infection.

As well as the sentence included in response to the previous point that more clearly identifies the existence of an effect of temperature on oocyst performance as a central result from the empirical data (“Although processes in natural systems are unlikely to map perfectly to tightly controlled experiments, this result suggests that oocysts are sensitive to temperature, and thus might have performance limits at higher and lower levels of temperature that could modulate overall transmission of the pathogen”), we have now also introduced the theme of biological limits on transmission associated with a lower and upper temperature:

“Since biological limits across abiotic variables like temperature are expected, we included both minimum and maximum temperatures.”

4) Incorrect statement about average age at infection (page 9).
The sentence “Increasing the force of infection or the rate at which susceptible individuals become infected increases the average age of the first infection” is incorrect. In fact, the average age at first infection is inversely related to the force of infection: a higher force of infection typically leads to a lower average age at first infection. Please correct this sentence.

Many thanks for catching this and apologies for the error! We have edited ‘increasing’ to read ‘reducing’.

Reviewer #2: The objective of the study is clearly stated in the Introduction and the rationale for the models used is clearly explained. The population used for the modelling was clearly described and appropriate for the aim of the study.

Thank you!

Figures 2, 3 and S3 are cited in this section. This seems the best place for Figure 2 but I am not convinced it is the best place for Figure 3 or Figure S3. The information in Figure 3, especially Figures 3a and S3, has come from the literature search outlined in Figure S1. Consider including it in the results.
Results of the searches are also included in the text of the methods when they should be in the results section.

Many thanks for this suggestion. We have worked to clarify the source and role of Figure S2 in the main text, and now include this in the 3rd paragraph in the Introduction, thus reclassifying it as Figure S1. We feel that this is the appropriate location for this figure as it reflects background information that motivated the study, rather than actual results of the study.

We include the first reference to the previous Figure S1 (now Figure S2) into the methods as it describes the flow used in the literature search in ways that felt useful for understanding the process at this stage in reading the manuscript. However, we are happy to move it to the results if the editor thinks this is more appropriate.

We also altered our representation of the data on temperature, limiting it to the range of values used for fitting; and moving it to the supplement to improve the clarity of Figure 3. This is now Figure S3, and also indicates the lack of correlation between minimum and maximum temperature in our data. Figure S4 represents the country specific effects for the statistical model; and we have added a Figure S5 reflecting the full span of the requested sensitivity analyses.

There is also a discrepancy between the number cited in the text (87) and the number in Figure S1 (92).

Corrected

Reviewer #3: In this article, the authors used data from a meta-analysis, supplemented it, and used other open-access data on demographic and climate projections to present what the burden of congenital toxoplasmosis in Africa could be in the future. The term “future” sound presumptuous, and a reformulation such as “based on demographic projections and scenarios of temperature change of plus or minus 3 degrees” would be more appropriate.

We have edited the title following suggestions:

“**Evaluating how demography and temperature increase might alter the future burden of congenital Toxoplasmosis in Africa**”

The approach and reasoning are interesting, but the discussion of uncertainties and biases inherent in both meta-analysis and climate and demographic model projections should be further developed. For example, all meta-analyses document the means of data collection (sources and search strategies), summarize them, and discuss the value of the primary outcome measure in search of factors that could affect both the reproducibility and validity of the conclusions. The same rigor would be appropriate here and would help to alleviate some concerns:

Thank you, following the reviewer’s suggestion we included details on the sources and search strategies of the meta-analysis in the methodology. The section reads as follows: “We leveraged a meta-analysis of seroprevalence across pregnant women (Gelaw et al. 2024), where authors searched through various databases to retrieve pertinent findings about the prevalence of toxoplasmosis infection in Africa using the following Mesh terms (“Toxoplasmosis” OR “Toxoplasma gondii”), AND (“Pregnant Women” OR “Prenatal care”), AND (“Africa”), as well as combinations including the names of individual African countries. We further expanded the dataset by conducting citation-based snowballing, in which we examined the references of included papers and subsequent studies citing them. Eligible studies reported seroprevalence in people and provided sufficient information on age groups, sample sizes, and prevalence estimates by age. We included studies that used any diagnostic method for detecting T. gondii antibodies (Supplementary Figure S2), restricting the search to those conducted in Africa since 1979 when detailed temperature data became available.”

- How was the search for additional articles conducted using the snowball method, and what elements made them relevant?

Thank you for pointing this out! We have edited the methods section to include a more detailed description of the snowball method, including,

“We further expanded the dataset by conducting citation-based snowballing, in which we examined the references of included papers and subsequent studies citing them. Eligible studies reported seroprevalence in people and provided sufficient information on age groups, sample sizes, and prevalence estimates by age. We included studies that used any diagnostic method for detecting T. gondii antibodies (Supplementary Figure S2), restricting the search to those conducted in Africa since 1979 when detailed temperature data became available.”

- What are the determinants and limitations of the open source upon demographic data? These elements should be considered in the discussion and general conclusion.

We now point to the documentation that provides a detailed overview of data sources, the strengths and limits and management of uncertainty in these projections.

“To capture this, we use estimates and forecasts provided by the UN Population Division, focussing on ‘Births by Age of Mother’ [[14]](https://paperpile.com/c/eFk1Tx/QwtY), which accounts for both shifts in age structure, and changing patterns of fertility over age (see XX for a discussion of how uncertainty is evaluated in these projections). Despite their broad utility, these data have important limitations. In many low- and middle-income countries, primary demographic inputs are sparse, outdated, or affected by underreporting. Therefore, our results should be interpreted as scenario-based projections rather than precise forecasts. To that effect, we included a section in the discussion that reads as follows: “	”

**Results**

Reviewer #1: The analysis follows the pre-specified plan.
However, there are several concerns regarding the results.

1) How was the temperature classification defined in Figure 3A?

We used the R function ‘colorRampPalette’ to generate 24 values spanning the colour range from blue to red. We then subtracted the lower range of our maximum temperature range (18 degrees celsius) from the range of maximum temperatures in our dataset, and indexed each of these temperatures to the colour ramp. For example, the first ‘blue-est’ colour is assigned to maximum temperature values that were closest to 19 degrees celsius, the second bluest colour to values closest to 20 degrees celsius, etc. We have now increased the point size and provided a continuous legend to try and make this more apparent. We also have extended the discussion of this future.

2) The authors selected studies targeting pregnant women. Could the authors clarify why a wide range of age groups is included in Figure 3A?

We thank the reviewer for raising this point. Figure 3A was intended to display the full range of available data, including all individual measurements extracted from the supplementary materials of the included studies, which resulted in the inclusion of a wide age range.

3) It would be preferable to arrange the countries in the same order as in Figure 5 for consistency and easier comparison.

We’re not entirely sure which point this refers to? In each instant countries have been organized according to magnitude of an effect. TODO change in we change up Figure 3?

Reviewer #2: The analysis presented in the results matches the analysis outlined in the methods and the results are clearly presented. The figures are mostly of good quality although some of the information is hard to see but clarity could be improved. It might be due to me looking at a pdf but colours were hard to distinguish and the font size seemed small. More specific comments:

Figure 2: the first half of the figure is very well explained but there is almost nothing the figure legend about the second half. There is also very little about it in the text as it as the Figure is cited as part of the methods. It is an important point to understand so I would recommend adding a couple more sentences.

Many thanks for this suggestion. We have considerably expanded the figure caption to try and make this clearer. It now reads:

“**Figure 2: Schematic indicating the paradoxical effect relating the force of infection (FOI) and burden of congenital toxoplasmosis,** assuming for simplicity that pregnancies start occurring in individuals aged > 19 years**.** As the force of infection increases (x axis, left hand side), the proportion of individuals seropositive by age 19 (y axis, left hand side) will also increase. For individuals living in a location with a high force of infection (blue) almost everyone will already have been infected by age 19 (left hand side, blue point at ~100% seropositive), which will mean that very few individuals will be at risk of acquiring a new infection during pregnancy (right hand side, blue point close to zero). Conversely for individuals living in location with a very low force of infection (green), very few individuals will be infected at all (left hand side, green point close to zero), so although many individuals remain susceptible by age 19, they are at little risk of acquiring the infection, and thus the risk of a first infection occurring during pregnancy is also low (right hand side, green point close to zero). Individuals living in settings with an intermediate force of infection (black) are potentially at the highest risk, as they are neither guaranteed to have already been infected (blue point), nor at very low risk (green point); thus, paradoxically, in high FOI settings, a decline in the FOI can result in an increase in burden depending on details of distribution of fertility”

Figures 3 and S3: More information is needed in the legend. Make it clear that A is total seroprevalence. Explain why Ghana was chosen as the representative for fertility trajectory. Is there a reason why the seroprevalence in pregnant women is not included in the main paper? I think it would add value since the paper is about risks of congenital toxoplasmosis. Also, the colours in Fig S3 don't match the colours in Fig 3a but there is no explanation of what the colours relate to in the legend.

We have expanded this legend as suggested. We do include pregnant women TODO: Fidy check? Also with Ingrid on description of how data was obtained?

Our original idea was the represent the full spectrum of the data available on this particular figure, but following the reviewers suggestions, it seems that this was not terribly useful, and thus we have chosen to remove the depiction of temperature ranges across countries, now including it in the supplement; and to remove the depiction of Ghana’s fertility altogether, as a more complete representation of fertility trajectories is now available in Figure 5.

Reviewer #3: Tables and graphs allow results to be visualized and guide discussions. Care should be taken to ensure they are clear, and priority should be given to those that are most relevant.

The results according to the different scenarios should be presented and discussed in greater detail and more clearly (conversely, Tables 1 and 2 could be provided as additional information).

We have reworked all of the Figures (as discussed in further detail in our response to the editor), and now also provide an extensive sensitivity analysis for greater clarity. We also moved the two Tables to the supplement as suggested (now Table S2 and Table S3).

Figure 3 is only briefly discussed (just the part A). When it comes to temperature variations, shouldn't we pay more attention to the range than to minimum or maximum values?

Many thanks for this suggestion. We have considerably simplified Figure 3, concentrating purely on the serology data. We now discuss across the manuscript in more detail why minimum and maximum temperatures are included; and included a justification that reads as follows:

“Since biological limits across abiotic variables like temperature are expected, we included both annual minimum and maximum of the daily mean temperatures. The variance inflation factor (calculated using the R package rvif) indicated that multicollinearity between these two variables was sufficiently small as to not destabilize the regression (V=1.08) as indicated in Figure S3.”.

Figure 5 is an example of an overloaded graph: the presentation of these data should be revised to make them more readable (and discussable), for example by dividing them according to temperature scenario and demographic scenarios. A map-based presentation should also be considered.

We have worked to clear up Figure 5, and these results are now complemented by the results from the suggested sensitivity analysis (Figure S5).

**Conclusions**

Reviewer #1: Authors clearly described the limitations of the study.

Reviewer #2: The conclusions are supported by the data presented and the authors have adequately addressed the limitations of the study. Even with the unavoidable limitations this study provides interesting results that will need to be considered by government authorities as they plan for a future where global temperatures rise.

Many thanks - and in this revision we have tried to bring out both limitations and planning potential in line with these suggestions and those of other reviewers more clearly.

Reviewer #3: Authors should formulate their results, comments, and conclusions with greater caution and reserve, partly because of biases and uncertainties (no work is entirely free of bias and as the X-Files series says, “the truth is out there”). On the other hand, care must be taken with the wording and the message that will be drawn from this work (we cannot suggest doing nothing to improve the HDI or the age of first pregnancy, so that the force of infection remains high and the risk of congenital toxoplasmosis remains low in the future). In addition, other hypotheses could be discussed by the authors: it is likely that temperature changes as significant as 3 degrees will cause radical changes to both ecosystems and economic systems and trigger significant population movements.

This is an excellent point. We have tried to bring this out throughout but paid particular attention at the end of the opening paragraphs of the discussion, now stating that:

“However, these results should be interpreted as an initial step towards integrating demographic, climatic and epidemiological processes, and there remains substantial “*avenues*” for refinement.re is much more to do to further ground these results. ”

In the second paragraph of the discussion, we run across a range of limitations and also specifically mention the point about the magnitude of temperature increase:

“Further experimental analyses of *T. gondii* oocyst responses to temperature alongside further observational studies, perhaps across targeted temperature gradients could help to address this. However it is also important to recognize that increases in temperature of the scale explored here are likely to lead to much wider ecosystem disruption, for which impacts are likely to be hard to predict.”

**Summary and General Comments**

Use this section to provide overall comments, discuss strengths/weaknesses of the study, novelty, significance, general execution and scholarship. You may also include additional comments for the author, including concerns about dual publication, research ethics, or publication ethics. If requesting major revision, please articulate the new experiments that are needed.

Reviewer #1: (No Response)

Reviewer #2: This is a well-written paper that is relatively easy to follow. The authors have given very good explanations of why they chose the parameters they did and put the results into context of changes to infectious diseases transmission as the climate changes.

Thank you.

Reviewer #3: This work is interesting in that it brings together data on the seroprevalence of toxoplasmosis in Africa and demographic projections, and incorporates assumptions about temperature changes to estimate the force of infection by toxoplasmosis and the consequences for congenital toxoplasmosis. However, these are only estimates and probabilistic models, the respective limitations of which should be clarified and discussed.

We have expanded the discussion to both underscore the limitations of our analysis and emphasize the importance of probing these results with further empirical data. In particular, we included a section in the discussion that reads as follows: However, these results should be interpreted as an initial step towards integrating demographic, climatic and epidemiological processes, and there remain substantial “avenues” for refinement.

A key component of our projections relies on demographic estimates provided by the XX. These data integrate observed census with model-based corrections to account for incomplete or biased reporting. As a result, our estimates should be interpreted as scenario based projections rather than precise forecasts.

We also included suggestions for follow-up studies in the discussion, specifically : ”Further experimental analyses of T. gondii oocyst responses to temperature alongside further observational studies, perhaps across targeted temperature gradients could help to address this.”

The initial data and treatment results should be presented more clearly and should provide more information for discussion.

We have worked to clarify this throughout our revision, and we hope that this is much improved in this version of the manuscript.

The comparison between a virus (rubella virus) and a parasite (Toxoplasma gondii) should either be discussed in more detail (discussion of their differences, complexity of the parasitic cycle and its still unknown information content) or omitted.

We do feel that the rubella example is a powerful one here - but really most around the effect of the age profile of risk. As suggested, we have tried to bring out some of the differences between these two pathogens more clearly on first mention of rubella.

“This dynamic set of possible outcomes for CT parallels projections for congenital rubella syndrome (CRS) associated with introduction of vaccination [22,23]. Rubella is a directly transmitted completely immunizing infection, and pre-vaccination, most rubella infections occur during childhood, and are relatively mild. Introduction of the vaccine at scales insufficient to achieve herd immunity, has the potential to slow down acquisition of immunity and shift susceptibility into reproductive ages, increasing incidence of CRS.”

In addition, the reality of a vaccine against toxoplasmosis in human medicine deserves to be discussed, particularly in light of Africa's economic capabilities.

Many thanks for this suggestion. A human vaccine against *T. gondii* would indeed be a tremendous boon for human health but as the reviewer points out needs to be discussed in light of Africa’s economic capabilities. We include a section in the discussion to address this which reads as follows:

“While no human vaccine is yet available, development efforts are ongoing (Zhang et al. 2022; Innes et al. 2019) and the eventual deployment of any vaccine would need to be guided by robust cost–benefit analyses to avoid unintended epidemiological consequences. Lessons from other systems highlight the importance of these economic considerations as they may shape the uptake and effectiveness in a future human T. gondii vaccination program. Although the development of a human vaccine against *T. gondii* would be transformative globally, its realistic implementation in Africa will also depend on sustained investment in research capacity, biotechnology infrastructure, and health-system strengthening. A One Health approach that coordinates across human, animal, and environmental health sectors will be critical to manage transmission risk holistically (Innes et al. 2019; Aguirre et al. 2019). Such strategies could include targeted vaccination of cats to reduce environmental contamination by limiting oocyst shedding (Bonačić Marinović et al. 2020).”
