# Supplementary material for: Evaluating how demography and temperature increase might alter the burden of congenital Toxoplasmosis in Africa
Source: PLoS Negl Trop Dis. 2026 Mar 6;20(3):e0014058. doi: 10.1371/journal.pntd.0014058 (PMC12974952; doi:10.1371/journal.pntd.0014058)
Supplement: Attachment — Submitted filename: Projecting Toxo- Response to reviewers 2.docx [file pntd.0014058.s011.docx]

Many thanks for the invitation to resubmit our manuscript. Below, we provide a response to each of the reviewer suggestions in blue.

**Reviewers' comments:**

Reviewer's Responses to Questions

**Key Review Criteria Required for Acceptance?**


As you describe the new analyses required for acceptance, please consider the following:


**Methods**


Reviewer #1: Concerns were comprehensively addressed.

Thank you

Reviewer #2: The authors have taken note and made most the suggested changes from myself and the other reviewers. I note that there are still results included in the methods section around the searches that were made to identify the seroprevalence data. This information should form the start of the Results section and I believe the associated figure (the PRISMA diagram) should be included as a proper figure rather than a supplementary one. It contains important data.

Following the reviewers’ suggestions, we moved the section describing the number of articles documenting Toxoplasma seroprevalence in Africa to the Results section. However, we elected to retain the PRISMA diagram as a supplementary figure because it primarily details the literature search and screening process rather than presenting substantive study findings. While it provides transparency regarding study identification and selection, it does not contribute directly to the analytical results or core conclusions of the manuscript. To ensure clarity and accessibility, we now clearly reference the PRISMA diagram in the main text at the beginning of the Results section, so readers can readily consult it if desired. We are of course happy to revise this if the editor feels otherwise.

Reviewer #3: The objectives of this article are clearly stated and the design of this original study appears to be sound. The statistical analyses performed appear to be correct and support the conclusions stated.

Thank you

**Results**


-Does the analysis presented match the analysis plan?

-Are the results clearly and completely presented?

-Are the figures (Tables, Images) of sufficient quality for clarity?

Reviewer #1: Concerns were comprehensively addressed, , though a few minor editorial details remain.
1) Figure 2: The Y-axis on the left plot is labeled "Prop serpositive by age 19," missing an 'o' in "seropositive".

Corrected, thank you. The corrected figure is shown below

2) Figure 3: The Y-axis label is misspelled as "Propoertion IgG Positive".

Corrected, thank you

Reviewer #2: There have been significant improvements in how the results are presented. This section is much easier to follow now.

Thank you

Reviewer #3: The analyses are as expected, but work remains to be done on the quality, clarity, and readability of the figures:
Figure 3 shows more colors than the legend, and the size of the dots suggests another parameter that is not shown. This creates a discrepancy between the figure and its presentation that needs to be corrected. Furthermore, the title of the vertical axis needs to be corrected.

Corrected, thank you. The corrected figure is shown above and now includes more colors for the temperature gradient and indication of size for dots (hdi). This is now indicated in the legend and caption of the figure.

**Fig 3: Seroprevalence data extracted from the literature**. Each data point indicates a combination of age class midpoint (x axis) and proportion seropositive (y axis). Points are coloured by maximum city temperature at time of study. Dot size is proportional to the Human Development Index (HDI) of the country at the time of the study. The concentration of warmer colours (indicative of higher temperatures) towards the bottom of the plot suggests a reduction on transmission associated with high temperatures as suggested by Dubey et al.

Figure 4, which consists of several graphs, shows an overlap and should have a legend associated with it.

Corrected, Thank you. The corrected figure is now shown below:

Figure 5 is also fragmented and of poor quality (the results for Eswatini are truncated, for example). The legend is unclear and the relationship between 2050 and 2100 should be explained in the text (why is there stagnation between 2050 and 2100 for Benin and a reversal of the relative burden between 2050 and 2100 for the countries shown below?).

Corrected, Thank you

All documents placed in supplementary material should include a title and caption so that they can be understood independently of the text (in particular the Figure S5 where many colors and parameters are not explained).

Thank you. We corrected the titles and captions for supplementary materials. In particular Fig S5 title and captions read as follows:

**Fig S5: Projected relative burden under temperature and demographic change scenarios.** Projected relative burden of Toxoplasma gondii infection under scenarios of no temperature change and increases of +1°C, +2°C, and +3°C in maximum (x-axis) and minimum (y-axis) temperatures for the years 2023, 2050, and 2100. Each panel represents a temperature-change scenario, and projections incorporate corresponding demographic changes for each time period. Colour scales are standardized across panels, with deeper red indicating a higher relative burden and lighter colours indicating a lower relative burden. Future demography has larger effects in countries with ongoing population growth.

**Conclusions**


-Are the conclusions supported by the data presented?

-Are the limitations of analysis clearly described?

-Do the authors discuss how these data can be helpful to advance our understanding of the topic under study?

-Is public health relevance addressed?

Reviewer #1: Concerns were comprehensively addressed. Thank you

Reviewer #2: The authors have responded well to the issues raised by the reviewers and this section is fine. Thank you

Reviewer #3: The conclusions are supported by the data and are written in a more objective manner, with greater attention paid to the limitations of the data and analyses, giving this study greater scope. Thank you

**Editorial and Data Presentation Modifications?**


Use this section for editorial suggestions as well as relatively minor modifications of existing data that would enhance clarity. If the only modifications needed are minor and/or editorial, you may wish to recommend “Minor Revision” or “Accept”.

Reviewer #1: (No Response)

Reviewer #2: I did notice some grammatical errors so it would be useful to get someone who hasn't viewed the paper frequently to proof-read it. They aren't major errors.

Reviewer #3: Minor revision : plase edit the figure and table.

**Summary and General Comments**


Use this section to provide overall comments, discuss strengths/weaknesses of the study, novelty, significance, general execution and scholarship. You may also include additional comments for the author, including concerns about dual publication, research ethics, or publication ethics. If requesting major revision, please articulate the new experiments that are needed.

Reviewer #1: Concerns were comprehensively addressed, though a few minor editorial details remain as described above.
It is ultimately the authors' choice, but moving Figure S6 to the main text (perhaps as Figure 5, preceding the current Figure 5) would help demonstrate the separate effects of climate change and demographic shifts. This adjustment would allow for a more mechanistic understanding of how temperature affects risk, independent of demographic shifts, before presenting the total projected disease burden.

Reviewer #2: (No Response)

Reviewer #3: no comments
